# Supplementary material for: Interest in long-acting injectable ART among adolescents with perinatally acquired HIV in Cameroon: Implications for implementation in developing countries
Source: PLOS Glob Public Health. 2026 May 15;6(5):e0006293. doi: 10.1371/journal.pgph.0006293 (PMC13178921; doi:10.1371/journal.pgph.0006293)
Supplement: Attachment — Submitted filename: PGPH_Response to Reviewers_26032026.pdf [file pgph.0006293.s007.pdf]

Yagai Bouba PhD
Chantal Biya International Reference Centre (CIRCB),
PO Box 3077, Yaoundé, Cameroon,
romeobouba@yahoo.fr
March 27, 2026

Editor-in-Chief

PLOS Global Public Health

Dear Editor,

We sincerely appreciate the thoughtful feedback and constructive suggestions provided by you and the reviewers, which have helped strengthen our study entitled "Interest in long-acting injectable ART among adolescents with perinatal HIV in Cameroon: implications for implementation in Africa." We have carefully revised the manuscript to improve clarity and to address all the concerns raised. Below, we provide detailed, point-by-point responses to each comment. Revised and newly added text in the manuscript is track changes.

Reviewer 1

Major Comments

1. **Interpretation of High Interest in LAI-ART**

   The reported level of interest (92.3%) is striking. As LAI-ART is not yet available locally, this likely reflects hypothetical acceptability. The authors should acknowledge this more explicitly and clarify what information (e.g., injection frequency, clinic visits, potential side effects) participants received before expressing interest.

Response: We thank the reviewer for this important comment. We agree that the high level of reported interest likely reflects hypothetical acceptability, given that LAI-ART is not yet available in our setting. We have revised the Methods section to clarify that, prior to assessing interest, participants were provided with brief information describing LAI-ART as an injectable treatment administered every 8 weeks at the follow-up unit (Lines 199-204).

2. Measurement of "Interest" and "Knowledge"

The operational definitions of interest and knowledge are pragmatic but limited. Clarification is needed on whether "interest" reflects firm intention versus general preference. The discussion should reflect

on how limited or superficial knowledge of LAI-ART may affect real-world uptake.

Response: We now explicitly acknowledge in the manuscript that this reported interest should be interpreted as hypothetical acceptability rather than actual willingness under real-world conditions, particularly as detailed information on aspects such as potential side effects and broader logistical considerations was not extensively covered (Lines 199-204). Sentence were added in the discussion about these aspects (lines 401-409)

3. Fear of Injection as a Key Predictor

Fear of injection is an important and policy-relevant finding. The authors should briefly justify the use of a single-item, non-validated measure and discuss how injection fear might be addressed through counselling or adolescent-friendly education during rollout.

Response: We thank the reviewer for this important remark. While this point was briefly addressed in the earlier version, we have now considered it more thoroughly in the revised manuscript. We have clarified in the Discussion that fear of injection was assessed using a brief single-item, non-validated question, intended as a pragmatic measure to capture participants perceived concern in a field-based survey rather than to provide a detailed clinical assessment of needle phobia.

We have also expanded the Discussion to indicate that injection-related concerns should be proactively addressed during implementation through adolescent-friendly counselling, clear education on the injection process and expected side effects, peer and caregiver support, and youth-friendly service delivery approaches. (lines 377- 383).

4. Generalizability

Most participants were recruited from urban clinics in one region. The limitations section should more clearly address potential selection bias and implications for adolescents in rural settings or those not engaged in care.

Response: Thank you for this important remark. While the study was conducted in the Centre region, which may limit national representativeness, we included participants from both urban and rural settings within this region, including the Mbalmayo health district. This has been described in the Methods section (lines 172-176). However, we agree that generalizability remains limited. We have therefore revised the limitations section to more clearly acknowledge potential selection bias related

to recruitment from a single region, and the implications for adolescents in other regions with different characteristics (lines 401-404)

Minor Comments

1. **Ensure consistency in viral suppression definitions (<50 vs <1000 copies/mL) and clarify their analytical use.**

Response: Thank you for this important comment. We have clarified the definitions used in the manuscript to ensure consistency. Specifically, we now distinguish between viral suppression (<1000 copies/mL), based on WHO programmatic criteria, and viral undetectability (<50 copies/mL), which reflects the clinical threshold commonly used for LAI-ART eligibility. We have also ensured consistent use of these definitions throughout the manuscript and clarified their respective roles in the analysis. (Lines 211- 214). This revised accordingly throughout the paper.

2. **Table 1 is informative but dense; minor simplification would improve readability.**

Response: We thank the reviewer for this helpful suggestion. Given the descriptive nature of the study, we aimed to provide a comprehensive characterization of the study population. However, to improve readability, we have simplified Table 1. Specifically, we removed the stratification of CD4 cell count (cells/mm^3) from the table and reported it only in the text (lines 257-261). In addition, we streamlined the presentation of binary variables (e.g., sex, living with biological parents, injection drug use, and experience of stigma) by removing redundant categories. We hope that the revised table is now clearer and easier to interpret.

3. Figures would benefit from brief interpretive captions.

Response: We thank the reviewer for this helpful suggestion. We have revised the figure legends to include brief interpretive captions highlighting the key findings (see Figure 1 and figure 2 captions in lines 278-283 and 287-290). We hope this improves the clarity and readability of the figures.

4. Minor typographical and formatting issues (e.g., spacing in "LAI-ART") should be corrected.

Response: We have checked and corrected minor typographical and formatting issues, including spacing in "LAI-ART.

5. PLOS generally encourages open data sharing; the authors may wish to briefly justify the "data available upon request" approach in light of ethical considerations involving

Response: Thank you for this important comment. We acknowledge the PLOS data sharing requirements and have revised the Data Availability statement accordingly. We have now provided a de-identified dataset and accompanying data dictionary as Supporting Information files (S1 Dataset and S1 Data Dictionary), ensuring that all data underlying the findings are fully accessible while protecting participant confidentiality (lines 435-437).

Ethics and publication considerations

Ethical approvals, assent and consent procedures, funding sources, and conflicts of interest are clearly reported and appropriate. I have no concerns regarding dual publication, research ethics, or publication ethics.

Response: We appreciate your time and careful review of these aspects.

Recommendation

This is a strong and timely manuscript with clear implications for adolescent HIV care and future LAI-ART implementation in LMICs. However, minor to moderate revision is required. Addressing the points above will further strengthen its clarity and impact.

Response: We sincerely thank the reviewer for the thorough review and insightful comments, which have greatly contributed to clarifying several aspects and improving the readability of our manuscript.

Reviewer 2

Major Comments

1. **Data Availability Statement:** There is a discrepancy that must be resolved before publication In the manuscript draft (page 3). the statement reads" Data is available upon request from the corresponding author. " However, the PLOS data Policy requires authors to make all data underlying the findings fully available without restriction. Please revise this statement to either deposit the data in a public repository or provide it within the supporting Information files. An "available upon request " statement is generally not compliant with PLOS policy unless there are documented ethical or legal restrictions, which are not mentioned here.

Response: Thank you for this important comment. We acknowledge the PLOS data sharing requirements and have revised the Data Availability statement accordingly. We have now provided a de-identified dataset and accompanying data dictionary as Supporting Information files (S1 Dataset and S1 Data Dictionary), ensuring that all data underlying the findings are fully accessible while protecting participant confidentiality. See lines 435-437

Minor Comments/Suggestions

1. **Introduction (Lines 87-92):** The sentence " Inconsistent adherence reflects limited access...all which erode the discipline required for daily dosing" is somewhat long and dense. Consider breaking it into two sentences for improved readability.

Response: The sentence in lines 96-99 has been split into two for improved readability. It now reads "Inconsistent adherence may reflect limited access to comprehensive services tailored for adolescents. This is further compounded by developmental factors such as growing autonomy, peer influence and continuing cognitive maturation that erode the discipline required for daily dosing".

2. **Methods (Line 17)** Please clarify the formula notation for standard sample size calculations.

Response: We have clarified the formula notation for standard sample size calculations in the methods section by using the standard superscript notations Z^2 and d^2 instead of the initial Z^2 and d^2. Other notations were clearly defined (Line 178).

3. **Results (Table 1) The footnote for a"" is missing from the table itself.** Please add the corresponding symbol (e.g.,*) next to "Age at disclosure" in the table to match footnote

Response: The footnote symbol *"a"* was added to Table 1 next to "Age at disclosure". However, to ensure clarity, we have replaced the *"a" symbol* with * in both the table and footnote. (Table 1)

4. **Discussion (Line 324-325):** The phrasing "nine in ten of Cameroonian APHI wanted LAI-ART" is slightly informal. Consider rephrasing to "Over 90% of Cameroonian APHI expressed interest in LAI-ART" for consistency with the abstract and results.

Response: We completely agree with this observation. This has been addressed accordingly (Lines 326- 327).

5. **Discussion (Lines 391-393)**: The limitation paragraph is good, but it could be strengthened by briefly noting that cross sectional design captures interest at a single point in time, and that interest might change as adolescents gain more information or as LAI-ART becomes available. This also links well to your call for longitudinal Implementation research.

Response: We appreciate this remark. We have now strengthened the limitations as recommended. It now reads "Additionally, the cross-sectional design and recruitment of participants from clinics within a single region may introduce selection bias and limit the generalizability of our findings, particularly for adolescents in other regions who may have different demographic, socio-economic and clinical characteristics. This design also captures interest at a single time point; this interest, based on limited information, may change as adolescents gain a more comprehensive understanding of LAI-ART or as it becomes available in practice. These findings therefore underscore the need for longitudinal implementation studies to assess how initial preferences translate into actual uptake once LAI-ART becomes available in routine care. (Lines 401- 409).

Conclusion:

The manuscript is technically sound, ethically rigorous, and makes a valuable contribution to the literature. Addressing the data availability statement and the minor comments above will strengthen the paper. I recommend Minor Revisions

Response: We sincerely thank the reviewer for the thorough evaluation of our manuscript and for the constructive and insightful comments. We have carefully addressed the data availability statement and all the minor comments raised. We believe that these revisions have strengthened the manuscript and improved its overall clarity and quality.

Dear Editor, we have carefully reviewed and addressed all major and minor comments from the reviewers. We thank the reviewers for their insightful feedback, which has significantly strengthened the manuscript and improved its clarity and readability. We hope that the revised version will meet your expectations and be suitable for publication.

We look forward to hearing from you,

Sincerely,

Yagai Bouba
