# Supplementary material for: Interest in long-acting injectable ART among adolescents with perinatally acquired HIV in Cameroon: Implications for implementation in developing countries
Source: PLOS Glob Public Health. 2026 May 15;6(5):e0006293. doi: 10.1371/journal.pgph.0006293 (PMC13178921; doi:10.1371/journal.pgph.0006293)
Supplement: Attachment — Submitted filename: Reviewer Report_PLOS ONE_ FINAL.docx [file pgph.0006293.s002.docx]

**Reviewer Report**

**Manuscript Number:** PGPH-D-25-03641

**Title:** Interest in long-acting injectable ART among adolescents with perinatally acquired HIV in Cameroon: Implications for implementation in developing countries

**Comments to the Authors**

Thank you for the opportunity to review this manuscript, which addresses an important topic: the potential role of long-acting injectable antiretroviral therapy (LAI-ART) for adolescents with perinatally acquired HIV in Cameroon. Your work contributes valuable insights to the ongoing discussions about expanding HIV treatment options in low- and middle-income countries. Below, I offer some observations and suggestions to strengthen the manuscript before publication.

**Scientific Validity**

The study tackles a genuine public health challenge, adherence difficulties

Among adolescents living with HIV, explore their openness to a promising alternative. The cross-sectional design is appropriate for this exploratory phase, and your sample of 248 adolescents is representative. The ethical approvals and consent procedures are clearly documented, reflecting careful attention to participant protection.

However, a few methodological aspects would benefit from clarification:

**Representativeness:** Recruiting participants from four Clinics in Cameroon’s Centre region may not capture the full diversity of experiences among adolescents nationally. Rural-urban differences, regional variations in HIV services, and cultural factors could influence both awareness of and interest in LAI-ART. I would encourage you to acknowledge this limitation more explicitly in your discussion and consider how your findings might apply or not apply to other settings.

**Measuring fear of rejection:** You assessed injection-related fear using a single question with three response options. While this provides a useful snapshot, fear of needles can be multifaceted, encompassing anticipatory anxiety, past traumatic experiences, or concerns about pain. A brief comment acknowledging this measurement limitation would strengthen your interpretation, particularly since fear emerged as a significant predictor of interest.

**Data Availability:** Your current statement (“data available upon request”) does not align with PLOS Open Data policy. To comply with journal requirements, please either deposit anonymized data in a public repository (e.g., Figshare, Dryad, or an institutional database) or provide a clear explanation of any ethical or legal restrictions that prevent public sharing. This is a critical requirement for publication.

**Interpretation:** Your conclusion that rollout should prioritize education and youth friendly counselling is reasonable and grounded in your findings. However, given the cross-sectional design, I would encourage more cautious language regarding causal relationships. Phrases suggesting that specific interventions “will” address barriers could be softened to indicate that they “may” or “are likely to “help; this better reflects the level of evidence your design can support.

**Clarity of Presentation**

The manuscript is generally well organized and readable. The abstract effectively summarizes the study’s purpose, methods, and key findings. Your tables and figures convey important patterns, though a few adjustments would enhance clarity:

- Define abbreviations (e.g., MDSD, VS) at first use in each major section (abstract, main text, figure legends) to assist readers who may dip into different parts of the manuscript.
- Table 1 headings could be streamlined by considering using clearer column labels and ensuring that percentage signs and denominators are consistently presented.
- Viral suppression thresholds appear in different places (VL <50 copies/mL for undetectable). Clarifying which threshold applies in each context would prevent confusion.

**Ethical Considerations**

The ethical conduct of this study appears sound. You have secured appropriate approvals, obtained parental consent and adolescent assent, and described confidentiality measures. The only substantive ethical concern relates to data sharing, as noted above. Once this is addressed, the ethical framework will be complete.

**Strengths of the Study**

- The focus on adolescents with perinatally acquired HIV, a population that faces unique challenges and is often underrepresented in research, is a genuine strength.
- Your sample size is robust, and achieving 97% of your target enrolment speaks to careful planning and community engagement.
- The topic is globally relevant and timely, with direct implications for policy and programmatic decisions as LAI-ART becomes more widely available.
- Involving adolescents themselves in identifying drivers of interest (reducing pill burden, improving adherence) centres the voices of those most affected.

**Areas for improvement**

- Generalizability is limited by the geographic scope of recruitment: this should be acknowledged more fully.
- The single-item measure of injection fear, while practical, may oversimplify a complex construct.
- Data sharing arrangements need to be brought into compliance with journal policy.
- Some interpretive claims extend beyond what a cross-sectional design can support: tempering these would strengthen scientific rigor.

**Recommendation**

**Major Revision**

This Manuscript addresses an important question and offers findings that could inform future implementation efforts. Worth attention to the points above, particularly around data availability, acknowledgement of limitations, and more cautious interpretation, it would make a valuable contribution to the literature. I look forward to seeing a revised version.

**Confidential Comments to the Editor**

This is a timely and relevant study that explores an important question: how adolescents with perinatally acquired HIV in Cameroon view long-acting injectable ART. The finding that 92% express interest despite only 30% prior awareness is striking and has clear programmatic implications. The authors have assembled a Substantial sample and engaged with the adolescent community, which adds credibility to their work.

However, I have several concerns that temper my enthusiasm:

**Generalizability:** The sample was drawn from four clinics in one region, and 84% of participants are from urban settings. Cameroon's adolescent HIV population is more diverse geographically, culturally, and in terms of service access. The manuscript would benefit from a more subtle discussion of how these factors might shape the findings and limit transferability.

**Measurement limitations:** Assessing injection fear with a single question is a notable weakness. Given that fear emerged as a significant predictor, a more robust measure would have strengthened confidence in this finding. I suggest the authors acknowledge this limitation and, if possible, note it as an area for future research.

**Data Sharing:** The current data availability statement does not meet PLOS requirements. This must be resolved before publication. Given that the study involves a vulnerable population, there may be legitimate ethical reasons for restricted access, but these need to be clearly articulated.

**Interpretive Caution:** The conclusions, while reasonable, occasionally overreach.

For instance, stating that rollout “should prioritise” certain strategies implies a level of evidentiary support that a cross-sectional study cannot provide. Framing these as implications or considerations would be more appropriate**.**
