# Supplementary material for: Interest in long-acting injectable ART among adolescents with perinatally acquired HIV in Cameroon: Implications for implementation in developing countries
Source: PLOS Glob Public Health. 2026 May 15;6(5):e0006293. doi: 10.1371/journal.pgph.0006293 (PMC13178921; doi:10.1371/journal.pgph.0006293)
Supplement: Attachment — Submitted filename: Confidential Comments to the Editor_PLOS ONE.docx [file pgph.0006293.s003.docx]

**Confidential Comments to the Editor**

This is a timely and relevant study that explores an important question: how adolescents with perinatally acquired HIV in Cameroon view long-acting injectable ART. The finding that 92% express interest despite only 30% prior awareness is striking and has clear programmatic implications. The authors have assembled a Substantial sample and engaged with the adolescent community, which adds credibility to their work.

However, I have several concerns that temper my enthusiasm:

**Generalizability:** The sample was drawn from four clinics in one region, and 84% of participants are from urban settings. Cameroon's adolescent HIV population is more diverse geographically, culturally, and in terms of service access. The manuscript would benefit from a more subtle discussion of how these factors might shape the findings and limit transferability.

**Measurement limitations:** Assessing injection fear with a single question is a notable weakness. Given that fear emerged as a significant predictor, a more robust measure would have strengthened confidence in this finding. I suggest the authors acknowledge this limitation and, if possible, note it as an area for future research.

**Data Sharing:** The current data availability statement does not meet PLOS requirements. This must be resolved before publication. Given that the study involves a vulnerable population, there may be legitimate ethical reasons for restricted access, but these need to be clearly articulated.

**Interpretive Caution:** The conclusions, while reasonable, occasionally overreach.

For instance, stating that rollout “should prioritise” certain strategies implies a level of evidentiary support that a cross-sectional study cannot provide. Framing these as implications or considerations would be more appropriate**.**
