# Supplementary material for: Interest in long-acting injectable ART among adolescents with perinatally acquired HIV in Cameroon: Implications for implementation in developing countries
Source: PLOS Glob Public Health. 2026 May 15;6(5):e0006293. doi: 10.1371/journal.pgph.0006293 (PMC13178921; doi:10.1371/journal.pgph.0006293)
Supplement: Attachment — Submitted filename: COMMENTS TO THE EDITOR Prt2_PLOS ONE.docx [file pgph.0006293.s006.docx]

**COMMENTS TO THE EDITOR**

The authors have comprehensively and respectfully addressed all concerns raised during the first round of peer review. Key improvements include clearer distinction between hypothetical interest vs. Actual willingness, transparent handling of measurement limitations (e.g., single item injection fear assessment), explicit strategies for implementation, and resolution of data availability and viral suppression definition issues. The manuscript is now methodologically sound. Ethically rigorous and policy relevant. No further peer review is required.
