# Supplementary material for: Interest in long-acting injectable ART among adolescents with perinatally acquired HIV in Cameroon: Implications for implementation in developing countries
Source: PLOS Glob Public Health. 2026 May 15;6(5):e0006293. doi: 10.1371/journal.pgph.0006293 (PMC13178921; doi:10.1371/journal.pgph.0006293)
Supplement: Attachment — Submitted filename: Reviewer Comments prt2_PLOS ONE_Comments to the Authors.docx [file pgph.0006293.s005.docx]

**Decision Recommendation: Accept** (no further peer review required)

**Comments to the Authors:**

Dear Dr. Bouda and colleagues,

Thank you for the careful and thoughtful revision of your manuscript. I have reviewed your point-by-point responses and the revised manuscript with tracked changes. You have addressed the concerns raised during the first round of review thoroughly and respectfully. The manuscript has improved substantially in clarity, transparency, and scientific rigor.

Below, I offer my final assessment of how each major comment from the previous round has been addressed, along with a few observations. No further revisions are required, and I am pleased to recommend acceptance.

Response to Major comments from the first review

- **Hypothetical acceptability of interest:** You have appropriately clarified in the Methods section (lines 204) that interest was assessed after participants received brief information about an injectable regimen administered every eight weeks, and you now explicitly acknowledge in the Discussion (lines 401-409) that this represents hypothetical rather than actual willingness. This addressed the concern well.
- **Definition of Interest and Knowledge:** The revised manuscript now distinguishes more clearly between stated preference and firm intention, and the limitations section appropriately notes that interest may evolve as adolescents gain more comprehensive information about side effects, logistical requirements, and long-term implications. This is a balanced and honest representation.
- **Fear of Injection Measurement:** You have provided a reasonable justification for the single-item, non-validated measure as a pragmatic field-based indicator. More importantly, you have expanded the Discission (lines 377-383) to include concrete, actionable strategies to mitigate injection fear during rollout, including adolescent-friendly counselling, peer and caregiver support, and youth-friendly service delivery. This strengthens the policy relevance of your findings.
- **Generalizability and selection bias:** The limitations section (lines 401-409) no longer clearly acknowledges potential selection bias due to recruitment primarily from one region (Centre region) and the predominance of urban clinics, while noting that rural representation was limited to one district hospital. This is appropriately cautious and does not overclaim the findings.
- **Consistency of viral suppression definitions:** You have now clearly distinguished between viral suppression (<1000 copies/ML) for programmatic purposes and viral undetectability. (<50 copies/mL) for LAI/ART eligibility. This is applied consistently throughout the manuscript, resolving the earlier confusion.
- **Table 1 readability:** The revised table is indeed clearer and less dense. Removing the CD4 stratification from the table while retaining it in the text was a sensible decision.
- **Figure Captions:** The legends for Figures 1 and 2 now include brief interpretive sentences, which improve accessibility for readers.
- **Data availability statement:** You have resolved the prior discrepancy by providing a de-identified dataset and data dictionary as supporting information files. This now fully complies with PLOS data-sharing policies.

In conclusion, I am satisfied that the authors have responded fully and appropriately to all concerns raised in the first round of review. The manuscript will be of interest to HIV programme managers, Paediatric and adolescent HIV clinicians, and implementation scientists working in low and middle-income countries. No further peer review is required from my side.
